# Reply to Martínez-Murcia et al. Reassessment of *Aeromonas oralensis*. Comment on “Mashzhan et al. Whole-Genome Sequencing of a Potentially Novel *Aeromonas* Species Isolated from Diseased Siberian Sturgeon (*Acipenser baerii*) Using Oxford Nanopore Sequencing. *Microorganisms* 2025, *13*, 1680”

**DOI:** 10.3390/microorganisms14051081

**Published:** 2026-05-11

**Authors:** Akzhigit Mashzhan, Izat Smekenov, Serik Bakiyev, Kalamkas Utegenova, Diana Samatkyzy, Asset Daniyarov, Ulykbek Kairov, Dos Sarbassov, Amangeldy Bissenbaev

**Affiliations:** 1 Scientific Research Institute of Biology and Biotechnology Problems, Al-Farabi Kazakh National University, Almaty 050040, Kazakhstan; 2 Almaty Branch of the National Center for Biotechnology, Central Reference Laboratory, Zhahanger St. 14, Almaty 050054, Kazakhstan; 3 Department of Molecular Biology and Genetics, Faculty of Biology and Biotechnology, Al-Farabi Kazakh National University, Almaty 050040, Kazakhstan; 4 Educational Programs for Training Teachers of Biology, Geography, Chemistry, Faculty of Natural Geography, Makhambet Utemisov West Kazakhstan University, Uralsk 090009, Kazakhstan; 5 Center for Life Sciences, National Laboratory Astana, Nazarbayev University, Astana 010000, Kazakhstan; 6 Faculty of Natural Sciences, L.N. Gumilyev Eurasian National University, 2 Kanysh Satpayev Street, Astana 010008, Kazakhstan

We thank the authors of the Comment [1] for their careful evaluation of our study and for highlighting important aspects of the taxonomic interpretation of strain AB005. Their remarks allow us to clarify our approach and place our findings within current taxonomic practice.

## 1. Phylogenetic Analysis

Based on multilocus sequence analysis, strain AB005 clusters within the *Aeromonas hydrophila* lineage. This result is supported by high bootstrap values and reflects its close relationship with other members of the *A. hydrophila* group.

However, phylogenies based on a limited number of genes may not distinguish fine-scale differences among closely related strains. Recent studies therefore recommend genome-scale approaches using larger sets of conserved markers. Methods such as GTDB-based analyses provide improved resolution and may further refine the position of strain AB005.

## 2. Genome Similarity Metrics

ANI values between strain AB005 and *A. hydrophila* strains exceed 96%, indicating close genomic relatedness. However, the dDDH value relative to the type strain (*A. hdrophila* ATCC 7966) is 68.9%, which is slightly below the reference value of 70%.

While these thresholds are widely used, they should be interpreted as guidelines, particularly in borderline cases. As the type strain is central to species delineation, the observed dDDH result suggests that strain AB005 lies near the boundary of the *A. hydrophila* species cluster.

## 3. Phenotypic Characteristics

We agree that biochemical traits may vary between individuals of *A. hydrophila*. The reactions reported for strain AB005 are within the known range of variability within the species.

In this study, phenotypic traits were not used as standalone criteria. Instead, they were evaluated alongside genomic data to support the characterization of the strain as a whole.

## 4. Taxonomic Framework

We acknowledge that the International Committee on Systematics of Prokaryotes (ICSP) is responsible for nomenclature, but not for defining species boundaries. The taxonomic literature provides criteria for species delineation, including recent recommendations by Chun et al. (2018) and Riesco and Trujillo (2024) [2,3].

Current practice emphasizes genome-based comparisons, supported by phylogenetic and phenotypic data. In this framework, borderline cases require careful interpretation rather than the strict application of thresholds.

## 5. Nomenclatural Aspects

We recognize the importance of the formal requirements necessary for the valid publication of new taxa, including the designation of a type strain and its deposition in a culture collection. These steps are essential for nomenclatural validity.

The present study focuses on characterizing strain AB005 and determining its taxonomic position. Full validation will require compliance with all nomenclatural criteria, and the study would benefit from the inclusion of additional isolates.

## 6. Interpretation of Strain AB005

Strain AB005 shows high genomic similarity to *Aeromonas hydrophila*, yet it also exhibits differences at genomic and phenotypic levels. The combination of ANI values above the threshold and a slightly reduced dDDH value relative to the type strain means this strain is in a borderline position.

Such intermediate cases are expected in closely related bacterial groups. In this context, strain AB005 can be considered a distinct lineage within the *A. hydrophila* complex.

## 7. Conclusions

Based on phylogenetic and genomic data, strain AB005 is closely related to *Aeromonas hydrophila*. However, the dDDH value relative to the type strain is slightly below the recommended threshold, and other distinguishing features have been identified.

Taken together, these results suggest that strain AB005 occupies a position near the boundary of the *A. hydrophila* species cluster. We therefore consider this strain to represent a differentiated lineage with potential taxonomic significance. Further comparative analyses and additional data will inform its final classification.
